# Characteristics and Driving Factors of the Aerobic Denitrifying Microbial Community in Baiyangdian Lake, Xiong’an New Area

**DOI:** 10.3390/microorganisms8050714

**Published:** 2020-05-11

**Authors:** Shilei Zhou, Yue Sun, Zaixing Li, Tinglin Huang

**Affiliations:** 1 Pollution Prevention Biotechnology Laboratory of Hebei Province, School of Environmental Science and Engineering, Hebei University of Science and Technology, Shijiazhuang 050018, China; hbkjdxsy@126.com; 2 Shaanxi Key Laboratory of Environmental Engineering, Xi’an University of Architecture & Technology, Xi’an 710055, China; huangtinglin@xauat.edu.cn

**Keywords:** aerobic denitrifying bacteria, MiSeq high-throughput sequencing technique, network analysis, *nap*A, nitrogen fractions, Baiyangdian Lake

## Abstract

Here, the ion-exchangeable form of nitrogen (IEF-N), weak-acid extractable form of nitrogen (WAEF-N), strong-alkali extractable form of nitrogen (SAEF-N), strong-oxidant extractable form of nitrogen (SOEF-N), residue nitrogen (Res-N), and total nitrogen (TN) showed spatial differences, and most of the sediment nitrogen fractions exhibited positive correlations in Baiyangdian Lake. High-throughput sequencing analysis revealed that the aerobic denitrification microbial community was composed of proteobacteria (42.04%–99.08%) and unclassified_bacteria (0.92%–57.92%). Moreover, the microbial community exhibited significant differences (R^2^ = 0.4422, *P* < 0.05) on the basis of the adonis analysis. T(temperature), Moisture content (MC), sediment total phosphorus (STP), ion-exchangeable form of ammonia (IEF-NH_4_^+^-N), weak-acid extractable form of ammonia (WAEF-NH_4_^+^-N), weak-acid extractable form of nitrate (WAEF-NO_3_^−^-N), and strong-alkali extractable form of ammonia (SAEF-NH_4_^+^-N) were the dominant environmental factors and explained 11.1%, 8.2%, 10.7%, 6.9%, 9.3%, 8.1%, 10.5%, 7.5%, and 7% variation, respectively, of the total variation in the microbial community. Furthermore, the network analysis showed that symbiotic relationships accounted for a major percentage of the microbial networks. The keystone aerobic denitrifying bacteria belonged to *Comamonas*, *Rhodobacter*, *Achromobacter*, *Aeromonas*, *Azoarcus*, *Leptothrix*_Burkholderiales, *Pseudomonas*, *Thauera*, unclassified_Burkholderiales, and unclassified_bacteria. The composition of the keystone aerobic denitrifying microbial community also exhibited significant differences (R^2^ = 0.4534, *P* < 0.05) on the basis of the adonis analysis. T, STP, IEF-NH_4_^+^-N, ion-exchangeable form of nitrate (IEF-NO_3_^−^-N), WAEF-NO_3_^−^-N, SAEF-NH_4_^+^-N, and TN were the dominant environmental factors that explained 8.4%, 6.2%, 4.6%, 5.9%, 5.9%, 4.5%, and 9.4% variation, respectively, of the total variation in the keystone aerobic denitrifying microbial community. The systematic investigation could provide a theoretical foundation for the evolution mechanism of the aerobic denitrifying microbial community in Baiyangdian Lake.

## 1. Introduction

Excessive nitrogen concentrations present problems for water quality and cause eutrophication [1]. An increase in the nitrogen load degrades water quality. In the past few years, bioremediation has become one of the most promising treatments because of its low maintenance cost, effective performance, and reduced environmental impacts [2,3]. However, there are different oxygen requirements for nitrification and denitrification in the traditional biological nitrogen removal process, which make the nitrogen removal impractical in natural aerobic waters. Fortunately, the first aerobic denitrifying bacterium (*Thiosphaera pantotropha*) [4] has been isolated, which can achieve nitrogen removal under aerobic conditions. Previous studies have shown that aerobic denitrification occurs in sea sediments [5], Wadden Sea [6], wetland [7], coastal sediments [8] and reservoirs [9,10]. Therefore, recently, many researchers have focused on nitrogen removal with aerobic denitrifying bacteria [11,12].

Currently, some full-scale experiments on bioaugmentation with aerobic denitrification bacteria have been conducted successfully for the remediation of wastewater, urban river, and river sediment. Such as, Duan et al. (2015) explored the nitrogen removal performance of halophilic heterotrophic nitrification-aerobic denitrification SF-16 in saline wastewater [13]; Du et al. (2017) investigated the variation of bacterial community structure in an aerobic denitrification reactor of industrial wastewater [14]; Sun et al. (2018) achieved the bioremediation of sediment through the aerobic denitrification agent in an urban river [1] and evaluated the mechanism of aerobic denitrifying bacteria [15]. Comparison of bioaugmentation in wastewater, urban rivers, and urban sediments has shown that there is limited information on the addition of inoculated bacteria or enhancement of indigenous aerobic denitrification bacteria in situ in natural water ecosystems. To the best of our knowledge, only our previous study not only investigated the effects of stratification and mixing on microbial community structure [16,17]; but also showed that indigenous aerobic denitrification bacteria can be enhanced based on WLA technology in drinking water reservoirs [18,19,20]. Furthermore, it is important to ascertain whether the introduced microbial strains can survive and remain active in natural water ecosystems. In order to isolate efficient aerobic denitrifying bacteria and achieve nitrogen removal through aerobic denitrification in a natural water ecosystem, it was necessary for us to analyze the characteristics and driving factors of an aerobic denitrifying microbial community, especially “keystone species”, the dominant environmental factor in a natural water ecosystem. Previous studies have shown that *nap*A is the biomarker of aerobic denitrifying bacteria [9,21]. Therefore, we investigated variations of aerobic denitrifying bacteria through Illumina MiSeq technology based on the aerobic denitrifying functional gene (*nap*A) in Baiyangdian Lake.

In this study, our objectives were to (1) investigate the characteristics of environmental factors; (2) examine the composition of the aerobic denitrifying microbial community and keystone operational taxonomic unit (OTU); (3) evaluate the differences in abundance, diversity, and community structure; (4) analyze the relationship between the microbial community structure and environmental driving factors; and (5) estimate the relative contributions of the environmental driving factors.

## 2. Material and Methods

### 2.1. Study Area and Sample Collection

**Figure 1:**
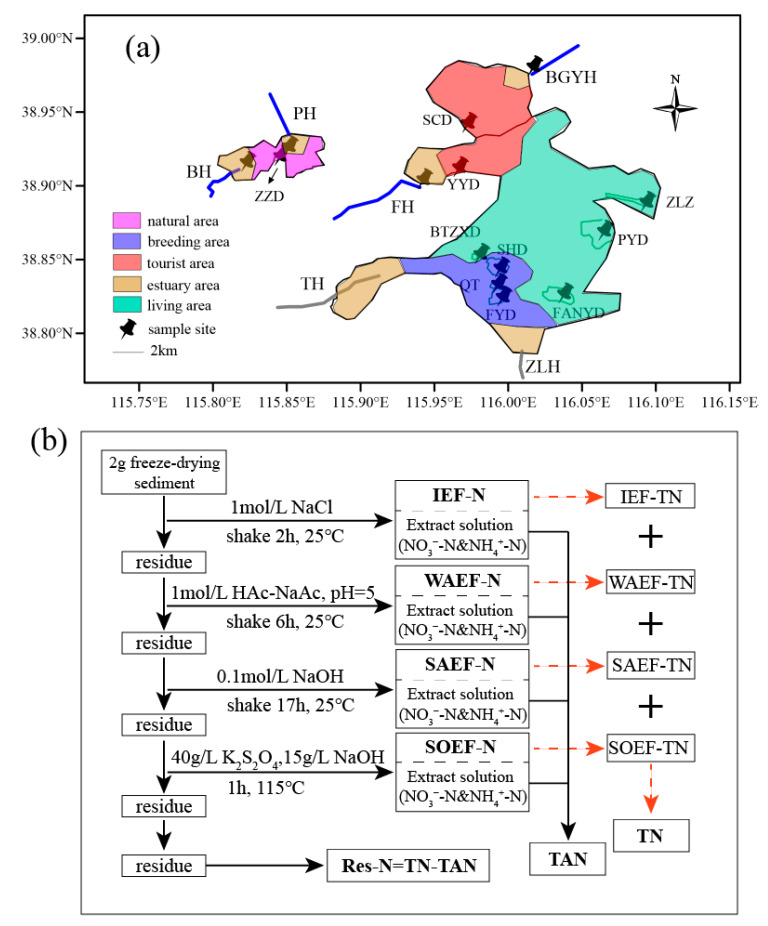
The sample sites and sequential extraction process of sediment N fractions in Baiyangdian Lake. (**a**), the map of sample sites; (**b**), the sequential extraction process of sediment N fractions.

Baiyangdian Lake, located in Xiong’an New Area, is the largest freshwater lake in the North China Plain (Figure 1a). Baiyangdian provides an important ecological guarantee for economic development in the surrounding areas. Baiyangdian Lake is divided into the tourist area, living area, natural area, breeding area, and estuary area. Water sampling was performed on January 15, 2019 with a 5-L surface-water sampler (1 m). The surface sediments (depth = 0–4 cm) were collected using a sterilized Petersen stainless steel grab sampler from 14 sites in Baiyangdian Lake (detail information is shown in the Supplementary Material). The samples were transported to our water research laboratory and stored in the dark at 4 °C.

### 2.2. Water and Sediment Quality Analyses

T, DO, pH, ORP, and EC of the water samples were determined using Hydrolab DS5 (Hach Company, USA). According to the standard methods, TN, NO_3_^−^-N, NO_2_^−^-N, and NH_4_^+^-N were measured using DR6000 (Hach Company, USA) [22]. The different forms of transferable nitrogen for sediment were evaluated using the sequential extraction method (Figure 1b) and more information is shown in the Supplementary Material [23,24].

### 2.3. Microbial DNA Extraction and MiSeq

The whole DNA of the sediment was extracted using the Soil DNA Kit (Omega, USA). After DNA purification, the extracted DNA was stored at −80 °C for PCR amplification. The extracted DNA was amplified using primer V66F, 5′-TAYTTYYTNHSNAARATHATGTAYGG-3′, and V67R, 5′- DATNGGRTGCATYTCNGCCATRTT-3′, for aerobic denitrifying bacterial *nap*A [9,25]. PCR amplification was performed as follows: 95 °C for 3 min; 40 cycles at 95 °C for 30 s, 55 °C for 30 s, and 72 °C for 45 s; and 72 °C for 10 min [9]. High-throughput sequencing was performed using the Illumina MiSeq platform and standard protocols at Shanghai Majorbio Bio-pharm Technology Co., Ltd. (Shanghai, China). Moreover, the sequencing data was deposited in the National Center for Biotechnology Information (NCBI, https://submit.ncbi.nlm.nih.gov/subs/sra/) database with the accession number PRJNA623955.

### 2.4. Microbial Community and Statistical Analysis

On the basis of the OTU data, the richness index, Shannon index, Simpson index, Pielou index, Chao1 index, ACE index, and Good’s Coverage were calculated to evaluate the alpha diversity. The differences in the microbial communities were evaluated using principal co-ordinate analysis (PCoA) and permutational MANOVA (adonis) in the vegan package (R.3.5.3). Redundancy analysis (RDA) was used to determine the correlation between the microbial community and predominant environmental factors with variance inflation factor (VIF) < 10 in the vegan package (R.3.5.3). Indicator species analysis was conducted using labdsv package (R.3.5.3). Hierarchical partitioning (HP) analysis was performed to quantitatively evaluate the relative influences of the environmental factors on the aerobic denitrifying bacterial community by using the rdacca.hp package (R.3.5.3) [26].

Network analysis was performed to investigate the biotic interactions between the microbial populations, and the results were visualized with Gephi software (0.9.2). In this study, a positive correlation implied a mutualistic interaction, whereas a negative correlation may indicate competition [27]. The “keystone OTUs” were recognized as OTUs within node degree > 5 and betweenness centrality value < 1000 [28].

## 3. Results and Discussion

### 3.1. Spatial Variation and Correlation Analysis of Sediment Nitrogen

**Figure 2:**
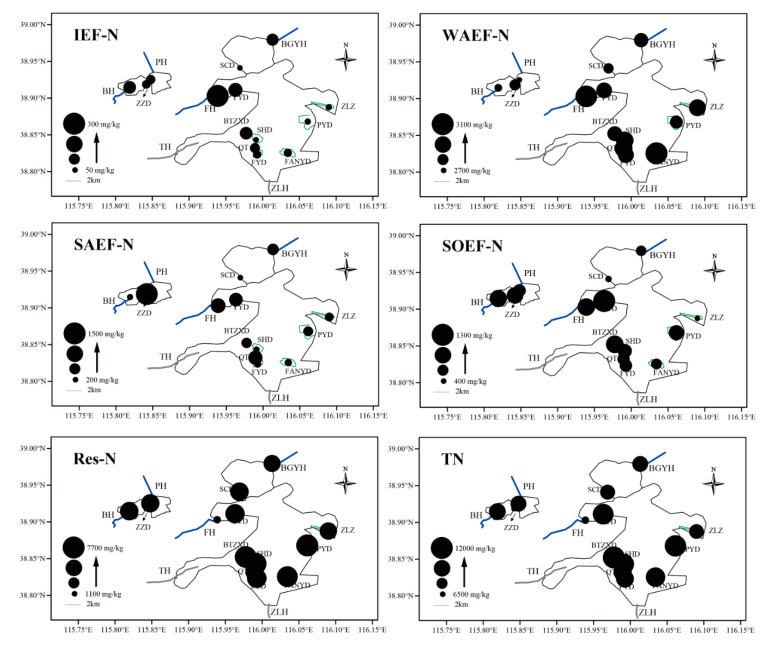
Spatial distribution for IEF-N, WAEF-N, SAEF-N, SOEF-N, Res-N and TN of sediment N in Baiyangdian Lake (the size of circle was proportional to the concentration of nitrogen).

**Natural area**. The IEF-N, WAEF-N, SAEF-N, SOEF-N, and Res-N values were 100.61, 2831.38, 1487.38, 995.42, and 1093.82 mg/kg, respectively (Figure 2). WAEF-N accounted for 43.50%, followed by SAEF-N (22.85%; Figure S1a). WAEF-NO_3_^−^-N reached 2693.23 mg/L, which accounted for 95.12% of WAEF-N. SAEF-NO_3_^−^-N and SOEF-NO_3_^−^-N accounted for 65.08% and 79.54% in SAEF-N and SOEF-N, respectively. **Breeding area**. The Res-N of FYD, QT, and SHD sample sites accounted for 61.89%, 56.44%, and 64.56% of the sediment nitrogen, respectively (Figure S1a). TAN values in the breeding area were in the order of QT > SHD > FYD. IEF-N, WAEF-N, SAEF-N, and SOEF-N ranged from 59.50 to 119.85, 2948.96 to 2996.46, 260.09 to 885.74, and 760.60 to 864.42 mg/kg, respectively (Figure 2). IEF-N, WAEF-N, SAEF-N, and SOEF-N accounted for 0.51%–1.10%, 25.39%–27.41%, 2.21%–8.10%, and 6.96%–7.34%, respectively (Figure S1a). **Tourist area**. The IEF-N, WAEF-N, SAEF- N, SOEF-N, and Res-N values were 57.03–176.78, 2814.33–2945.10, 206.59–841.28, 430.98– 1295.58, and 6308.96–6600.33 mg/kg, respectively (Figure 2). Moreover, the nitrogen content in the YYD sample site was obviously higher than that in the SCD sample site. **Estuary area**. The TAN proportion in the FU sample site was the highest among the sample sites of the estuary area and accounted for 74.48% of sediment TN. The IEF-N, WAEF-N, SAEF-N, SOEF-N, and Res-N ranged from 109.41 to 288.84, 2701.38 to 3084.66, 244.85 to 931.81, 657.87 to 1048.25, and 1834.42 to 6278.52 mg/kg in the estuary area (Figure 2), and the proportions were 1.07%–4.02%, 26.31%–42.91%, 2.34%–12.96%, 6.49%–14.58%, and 25.52%–61.10%, respectively (Figure S1a). **Living area**. The IEF-N, WAEF-N, SAEF-N, SOEF-N, and Res-N values were 96.08 ± 42.81, 2967.11 ± 92.39, 479.50 ± 109.01, 757.91 ± 295.29, and 7091.55 ± 839.31 mg/kg in the living area (Figure 2), and the proportions were 0.83% ± 0.31%, 26.29% ± 3.21%, 4.21% ± 0.85%, 6.50% ± 2.04%, and 62.17% ± 1.55%, respectively (Figure S1a).

**Figure 3:**
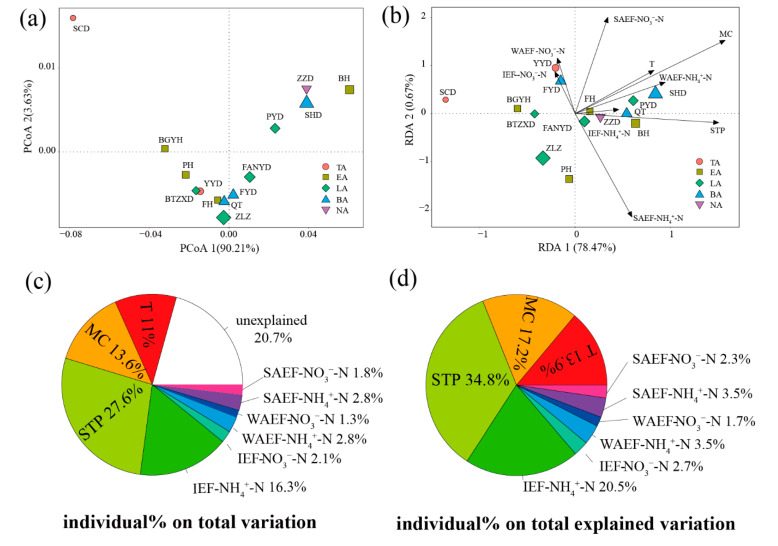
PCoA, RDA, and HP of aerobic denitrifying microbial community *α*-diversity in Baiyangdian Lake. (**a**), PCoA of aerobic denitrifying microbial community *α*-diversity; (**b**), RDA of aerobic denitrifying microbial community *α*-diversity; (**c**)(**d**), HP of aerobic denitrifying microbial community *α*-diversity.

WAEF-NO_3_^−^-N was positively correlated with Res-N (R = 0.38, *p* < 0.05), whereas it was negatively correlated with IEF-NO_3_^−^-N (R = −0.65, *p* < 0.001), SAEF-NH_4_^+^-N (R = −0.34, *p* < 0.05), IEF- N (R = −0.41, *p* < 0.01), and IEF-NH_4_^+^-N (R = −0.28, *p* < 0.05) (Figure S1b). Res-N was negatively correlated with SAEF-NH_4_^+^-N (R = −0.77, *p* < 0.001), IEF-N (R = −0.47, *p* < 0.01), SAEF-N (R = −0.75, *p* < 0.001), IEF-NH_4_^+^-N (R = −0.47, *p* < 0.01), SAEF-NO_3_^−^-N (R = −0.60, *p* < 0.001), and TAN (R = −0.60, *p* < 0.001) (Figure S1b). WAEF-NH_4_^+^-N was positively correlated with WAEF-N (R = 0.84, *P* < 0.001) and IEF-NO_3_^−^-N (R = 0.48, *p* < 0.01). IEF-NO_3_^−^-N was positively correlated with IEF-N (R = 0.53, *p* < 0.01) (Figure S1b). SAEF-NH_4_^+^-N was positively correlated with SAEF-N (R = 0.74, *p* < 0.001), SAEF-NO_3_^−^-N (R = 0.47, *p* < 0.001), and TAN (R = 0.48, *p* < 0.01) (Figure S1b). IEF-N was positively correlated with SAEF-N (R = 0.39, *p* < 0.05), IEF-NH_4_^+^-N (R = 0.98, *p* < 0.001), SAEF-NO_3_^−^-N (R = 0.40, *p* < 0.05), TAN (R = 0.66, *p* < 0.01), SOEF-NO_3_^−^-N (R = 0.60, *p* < 0.01), SOEF-N (R = 0.59, *p* < 0.05), and SOEF-NH_4_^+^-N (R = 0.44, *p* < 0.05) (Figure 3b). SAEF-N was positively correlated with IEF-NH_4_^+^-N (R = 0.39, *p* < 0.05), SAEF-NO_3_^−^-N (R = 0.94, *p* < 0.001), and TAN (R = 0.87, *p* < 0.001) (Figure S1b). IEF-NH_4_^+^-N was positively correlated with SAEF-NO_3_^−^-N (R = 0.39, *p* < 0.05), TAN (R = 0.67, *p* < 0.001), SOEF-NO_3_^−^-N (R = 0.64, *p* < 0.01), SOEF-N (R = 0.63, *p* < 0.01), and SOEF-NH_4_^+^-N (R = 0.50, *p* < 0.05) (Figure S1b). SAEF-NO_3_^−^-N was positively correlated with TAN (R = 0.91, *p* < 0.001), SOEF-NO_3_^−^-N (R = 0.53, *p* < 0.05), SOEF-N (R = 0.54, *p* < 0.05), and SOEF-NH_4_^+^-N (R = 0.47, *p* < 0.05) (Figure S1b). TAN was positively correlated with SOEF-NO_3_^−^-N (R = 0.76, *p* < 0.01), SOEF-N (R = 0.76, *p* < 0.01), and SOEF-NH_4_^+^-N (R = 0.60, *p* < 0.01) (Figure S1b). SOEF-NO_3_^−^-N was positively correlated with SOEF-N (R = 0.99, *p* < 0.001) and SOEF-NH_4_^+^-N (R = 0.77, *p* < 0.001) (Figure S1b). SOEF-N was positively correlated with SOEF-NH_4_^+^-N (R = 0.86, *p* < 0.001).

### 3.2. Overview of the Microbial Communities

After quality trimming, a total of 223754 sequences with an average length of 373 bp were obtained for the 14 sediment samples. MiSeq revealed a total of 1886 OTUs with 97% similarity (Table S1). The Shannon, Simpson, Pielou, richness, ACE, and Chao1 indexes exhibited significant differences in the estuary area, and those at the BH sample site were minimum values (2.71, 0.58, 0.34, 259, 292.55, and 304.66, respectively). The Shannon, Simpson, Pielou, richness, ACE, and Chao1 indexes for the breeding area were 5.88 ± 0.23, 0.96 ± 0.01, 0.68 ± 0.02, 389 ± 27.5, 483.86 ± 42.37, and 481.45 ± 41.66, respectively; those for the tourist area were 6.16–6.76, 0.97, 0.71–0.73, 391–599, 513– 762, and 510–766, respectively; those for ZLZ presented maximum values: 7.05, 0.98, 0.75, 708, 797.65, and 778.93, respectively; and those for ZZD exhibited low richness and diversity: 3.80, 0.79, 0.48, 254, 290.26, and 292.99, respectively. The average coverage for the 14 sample sites was higher than 0.99, which could reflect the real information of the microbial community [29].

PCoA1 and PCoA2 accounted for 90.21% and 3.63%, respectively. The distribution of *α*-diversity was influenced by PCoA1 (Figure 3a). Moreover, T, MC, STP, IEF-NH_4_^+^-N, IEF-NO_3_^−^-N, WAEF- NH_4_^+^- N, WAEF-NO_3_^−^-N, SAEF-NH_4_^+^-N, and SAEF-NO_3_^−^-N were important environmental parameters for alpha diversity on the basis of the VIF analysis (VIF < 10; Table S2). The RDA showed that RDA1 and RDA2 accounted for 78.47% and 0.67%, respectively (Figure 3b). RDA1 was mainly influenced for the variation of *α*-diversity, and T (R = 0.66), MC (R = 0.70), STP (R = 1.00), IEF-NH_4_^+^-N (R = 0.97), and WAEF-NH_4_^+^-N (R = 0.80) were the dominant environmental parameters (Table S2). The relative influences of dominant physicochemical factors on the diversity of the sediment aerobic denitrifying microbial community [30] were evaluated using HP analysis (Figure 3c,d). STP showed the greatest relative influence on the total variation in *α*-diversity, which accounted for 27.6%; IEF-NH_4_^+^-N, MC, T, IEF-NO_3_^−^-N, WAEF-NH_4_^+^-N, WAEF-NO_3_^−^-N, SAEF-NH_4_^+^-N, and SAEF-NO_3_-N, 16.3%, 13.6%, 11%, 2.1%, 2.8%, 1.3%, 2.8%, and 1.8%, respectively. Moreover, STP, IEF-NH_4_^+^-N, MC, and T accounted for 34.8%, 20.5%, 17.2%, and 13.9% of the total explained variation in *α*-diversity, respectively.

### 3.3. Microbial Community Composition

**Figure 4:**
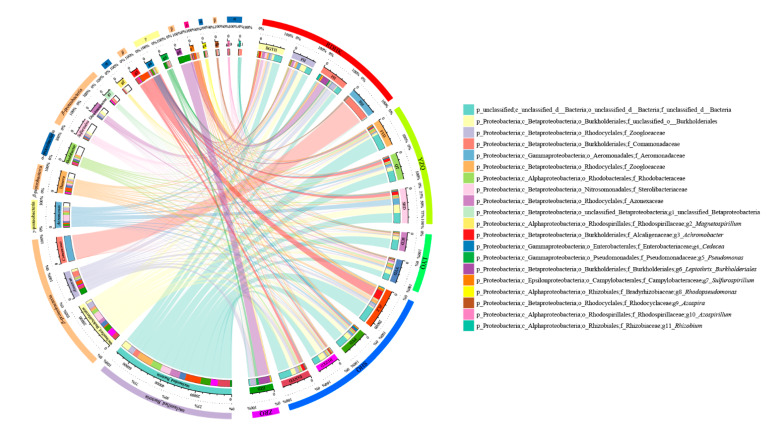
Distribution of aerobic denitrifying microbial community for each sample in the Baiyangdian Lake. (The width of the bars from each taxonomy indicate the relative abundance of that taxonomy in the sample).

At the phylum level, three main bacterial populations were detected, namely, proteobacteria (42.04%–99.08%), unclassified_bacteria (0.92%–57.92%), *Deinococcus-Thermus* (0.00–0.25%) (Figure 4). Proteobacteria at the ZZD sample site accounted for 93.84%, whereas that at the FYD sample site, 42.04% (minimum). A previous study showed that the phylum proteobacteria plays an important role in carbon and nitrogen metabolism [20,31].

At the class level (Figure 4), Betaproteobacteria was the largest class in Baiyangdian Lake and ranged from 21.10% to 86.65%. The percentage of Betaproteobacteria was maximum (86.65%) at the BH sample site and minimum at FYD (21.10%). For the estuary area, the percentages of betaproteobacteria ranged from 33.66% (PH) to 86.65% (BH); for the breeding area, 21.10% (FYD) to 50.50% (SHD); for the tourist area, 34.09% (SCD) to 57.25% (YYD); for the living area, 25.74% (ZLZ) to 49.18% (BTZXD); and for the natural area, 26.99%. Furthermore, species that belong to betaproteobacteria can degrade nitrogen [32], and they have been widely detected in various biotreatment systems, such as domestic wastewater [33] and cooking wastewater [34]. The percentages of alphaproteobacteria, gammaproteobacteria, and epsilonproteobacteria were 3.91–55.42%, 5.81–25.69%, and 0.01–7.51%, respectively; other major classes were unclassified_bacteria (0.92–57.92%), Deinococci (2.80–40.02%), and deltaproteobacteria (0–0.02%).

At the genus level, the top 20 abundant genera are as follows: *Comamonas* (0–83.37%), unclassified_bacteria (1.44–58.39%), *Rhodobacter* (0.01–50.84%), unclassified_Burkholderiales (0.46–28.84%), *Aeromonas* (0.08–23.42%), *Azoarcus* (1.19–22.78%), *Thauera* (0.31–18.17%), *Achromobacter* (0.02–11.86%), *Pseudomonas* (0.11–10.78%), *Sulfuritalea* (0.09–9.75%), unclassified_betaproteobacteria (0.28–9.28%), *Cedecea* (0–8.16%), *Dinoroseobacter* (0–7.61%), *Sulfurospirillum* (0–7.54%), *Leptothrix*_Burkholderiales (0.29–5.61%), *Magnetospirillum* (0.19–4.59%), *Azospirillum* (0.03–3.52%), *Azospira* (0–3.30%), *Rhodopseudomonas* (0.16–3.09%), and *Rhizobium* (0–2.10%) (Figure 4). Moreover, for the estuary area, the dominant genera were *Comamonas* (BH), unclassified_Betaproteobacteria (BGYH), *Achromobacter* (BGYH), *Sulfurospirillum* (PH), *Azospira* (BGYH), and *Azospirillum* (PH); for the breeding area, unclassified_bacteria (FYD) and *Thauera* (SHD); for the tourist area, *Sulfuritalea* (YYD) and *Dinoroseobacter* (YYD); for the living area, unclassified_Burkholderiales (BTZXD), *Aeromonas* (ZLZ), *Magnetospirillum* (ZLZ), *Cedecea* (PYD), *Leptothrix*_Burkholderiales (BTZXD), and *Rhizobium* (BTZXD); and for the natural area, *Azoarcus* (ZZD), *Rhodobacter* (ZZD), *Pseudomonas* (ZZD), and *Rhodopseudomonas* (ZZD). Previous studies have shown that these dominant genera are aerobic denitrifying bacteria. For example, *Comamonas* is capable of heterotrophic nitrification-aerobic denitrification nitrogen removal, and it has been successfully used for bioaugmentation of municipal wastewater [35] and enriched in a simultaneous nitrification and denitrification process [36]. Liu et al. (2018) showed that *Rhodobacter* is a typical denitrifying bacterium in alkaline copper mine drainage [37]. Aerobic denitrified species that belong to Burkholderiales were dominant, and they could remove nitric oxide after biomass combustion through nitrification and denitrification simultaneously in a membrane biofilm reactor [38]. Chen et al. (2014) investigated the impact resistance of extreme pH, low temperature, heavy metals, and high salinity on ammonia removal by the heterotrophic nitrifying-aerobic denitrifying bacterium *Aeromonas* sp. HN-02 [39]. Fu et al. (2019) found that aerobic denitrifying bacteria (*Aeromonas*, *Pseudomonas*, and *Acinetobacter*) could be successfully enriched and conducted denitrification to remove 90% of TN in a wetland test system [40]. *Azoarcus* was an important genus in a denitrifying quinoline-removal bioreactor [41], and it played an important role in high nitrate-removal performance under high salinity conditions in an airlift reactor [42]. *Thauera* was identified as the key potential heterotrophic nitrification and aerobic denitrification genus for principal nitrogen removal in granular reactors [43]. The dynamic central metabolic pathways of *Achromobacter xylosoxidans* CF-S36, a typical heterotrophic nitrification-aerobic denitrification bacterium, were investigated through molecular analysis [44]. Guo et al. (2018) evaluated the cold adaptation mechanism of a novel heterotrophic nitrifying and aerobic denitrifying-like bacterium, *Pseudomonas indoloxydans* YY-1, through physiological and transcriptomic analyses [45] and Yang et al. (2019a) investigated the simultaneous removal of nitrogen and phosphorous by heterotrophic nitrification-aerobic denitrification of a metal-resistant bacterium, *Pseudomonas putida* strain NP5 [46]. *Azospira* was the main dentrifier in a tidal flow constructed wetland [47], reservoir [9], and packed bed reactor [48]. *Halomonas* was the dominant heterotrophic nitrifying/aerobic denitrifying genus under hypersaline conditions [49]. *Rhodopseudomonas* is a potential autotrophic denitrifying genus that can use electrons and reducing power from cathodes [50]. Thus, the top 20 genera are all well-known aerobic denitrification bacteria, and they exhibited significantly spatial differences in Baiyangdian Lake. To investigate the evaluation mechanism of the aerobic denitrifying microbial community and isolate efficient aerobic denitrifying bacteria, unclassified_bacteria in Baiyangdian Lake need to be studied in the future.

### 3.4. Comparative Analysis and Driving Factors of the Microbial Community

Differences in the composition of the aerobic denitrifying microbial community in the five areas were investigated with PCoA and NMDS analysis. RDA was used to demonstrate the links between the environmental parameters and microbial community. HP analysis was performed to investigate the relative influence of the environmental driving factors on microbial community composition.

**Figure 5:**
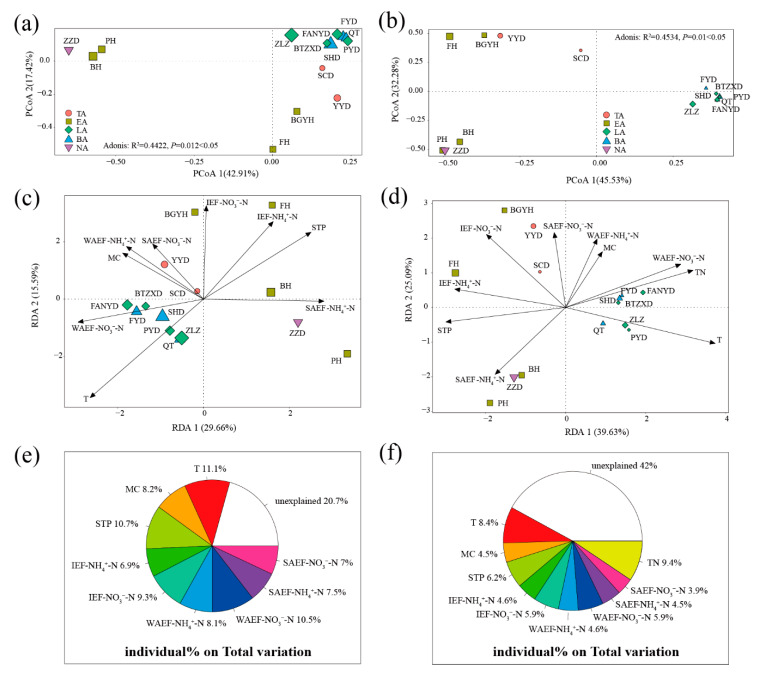
PCoA, RDA, and HP of whole and keystone aerobic denitrifying microbial community in Baiyangdian Lake. (**a**,**b**), PCA of whole and keystone aerobic denitrifying microbial community; (**c**,**d**), RDA of whole and keystone aerobic denitrifying microbial community; (**e**,**f**), HP of whole and keystone aerobic denitrifying microbial community.

**For the whole aerobic denitrifying microbial community**. The PCoA results showed that PCoA1 and PCoA2 accounted for 42.91% and 17.42%, respectively, of the variability in the microbial community composition (Figure 5a). Moreover, the aerobic denitrifying microbial community composition exhibited significant differences (R^2^ = 0.4422, *p* = 0.012 < 0.05) on the basis of the adonis analysis. The stress result (stress = 0.06 < 0.1) of NMDS showed that the NMDS analysis exhibited good representation (Figure S2a). On the basis of the PCoA and NMDS analysis results, samples from the same area clustered together, except for the estuary area; this suggested that the microbial community composition exhibited huge differences. On the basis of VIF (VIF < 10; Table S3), environmental parameters T, MC, STP, IEF-NH_4_^+^-N, IEF-NO_3_^−^-N, WAEF-NH_4_^+^-N, WAEF-NO_3_^−^-N, SAEF-NH_4_^+^-N, and SAEF-NO_3_^−^-N were important environmental factors for the whole aerobic denitrifying microbial community (Figure 5c and Table S3). RDA1 and RDA2 accounted for 29.66% and 15.59%, respectively, of the whole variation in the aerobic denitrifying microbial community, and RDA1 had a major influence on the variation. The samples from the breeding and living areas were located in quarter3; samples from the tourist area, quarter2; and samples from the natural and estuary areas, the positive side of RDA1. Moreover, T, MC, STP, IEF-NH_4_^+^-N, WAEF-NH_4_^+^-N, WAEF- NO_3_^−^- N, and SAEF-NH_4_^+^-N were the dominant environmental factors, and the correlation with RDA1 was −0.61, −0.76, 0.72, 0.53, −0.69, −0.95, and 1.00, respectively. T, MC, STP, IEF-NH_4_^+^-N, IEF-NO_3_^−^-N, WAEF-NH_4_^+^-N, WAEF-NO_3_^−^-N, SAEF-NH_4_^+^-N, and SAEF-NO_3_^−^-N explained 11.1%, 8.2%, 10.7%, 6.9%, 9.3%, 8.1%, 10.5%, 7.5%, and 7% variation of the total variation in the whole aerobic denitrifying microbial community (Figure 5e).

**For the keystone aerobic denitrifying microbial community**. The PCoA results showed that PCoA1 and PCoA2 accounted for 45.53% and 32.28%, respectively, of the whole variation in the composition of the keystone aerobic denitrifying microbial community (Figure 5b). Moreover, the aerobic denitrifying microbial community composition exhibited significant differences (R^2^ = 0.4534, *p* = 0.01 < 0.05) on the basis of the adonis analysis. The stress result (stress = 0.04 < 0.05) of NMDS showed that NMDS analysis exhibited excellent representation (Figure S2b). Moreover, samples from the same area, except for the estuary area, clustered together. On the basis of VIF (VIF < 10), T, MC, STP, IEF-NH_4_^+^-N, IEF-NO_3_^−^-N, WAEF-NH_4_^+^-N, WAEF-NO_3_^−^-N, SAEF-NH_4_^+^-N, SAEF-NO_3_-N, and TN were important environmental factors for the keystone aerobic denitrifying microbial community (Table S3). RDA1 and RDA2 accounted for 39.63% and 25.09%, respectively, of the whole variation in the keystone aerobic denitrifying microbial community (Figure 5d). Moreover, the samples from the breeding and living areas were located on the positive side of RDA1, whereas those from the tourist, natural, and estuary areas were located on the negative side of RDA1. T, STP, IEF-NH_4_^+^-N, IEF- NO_3_^−^-N, WAEF-NO_3_^−^-N, SAEF-NH_4_^+^-N, and TN were the dominant environmental factors, and the correlation with RDA1 was 0.96, −0.99, −0.98, −0.69, 0.93, −0.68, and 0.96, respectively. T, MC, STP, IEF-NH_4_^+^-N, IEF-NO_3_^−^-N, WAEF-NH_4_^+^-N, WAEF-NO_3_^−^-N, SAEF-NH_4_^+^-N, SAEF-NO_3_-N, and TN explained 8.4%, 4.5%, 6.2%, 4.6%, 5.9%, 4.6%, 5.9%, 4.5%, 3.9%, and 9.4% variation of the total variation in the keystone aerobic denitrifying microbial community (Figure 5f).

### 3.5. Network Patterns Revealed Microbial Community Characteristics

**Figure 6:**
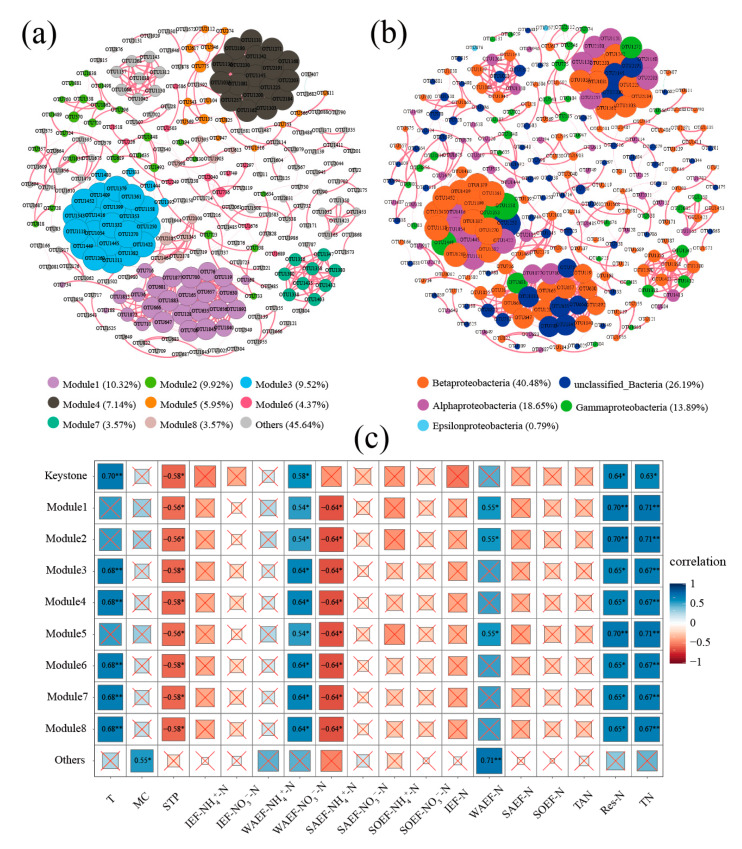
Network visualizes the OTU–OTU interactions and correlation between module and environmental factors in Baiyangdian Lake. Positive correlations were displayed in red and negative correlations were displayed in green (Spearman’s |R| > 0.9, *p*-value < 0.001). The size of each node is proportional to the degree. (**a**), OTU–OTU interactions (The nodes were colored according to modularity classes.); (**b**), OTU–OTU interactions (The nodes were colored according to class level.); (**c**), correlation between module and environmental factors.

Network analysis of the aerobic denitrifying microbial community was performed in Baiyangdian Lake (Figure 6). The network was composed of 252 nodes and 767 edges (Figure 6a). The network was colored by the top eight modularity classes and other modules: 10.32% (module 1), 9.92% (module 2), 9.52% (module 3), 7.14% (module 4), 5.95% (module 5), 4.37% (module 6), 3.57% (module 7), 3.57% (module 8), and 45.64% (9–46 modules). Moreover, the network was colored by class, and betaproteobacteria accounted for the maximum proportion in the network: Betaproteobacteria (40.18%), unclassified_bacteria (26.19%), alphaproteobacteria (18.65%), gammaproteobacteria (13.89%), and epsilonproteobacteria (0.79%) (Figure 6b). The network analysis showed the positive edges accounted for 99.87%, which indicates that symbiotic relationships accounted for a major proportion of the microbial networks. The keystone OTUs included 83 OTUs and belonged to module 1 (23 OTUs), module 2 (1 OTU), module 3 (22 OTUs), module 4 (18 OTUs), module 5 (1 OTU), module 7 (9 OTUs), module 8 (1 OTU), and others (8 OTUs). Furthermore, these keystone OTUs belonged to *Achromobacter*, *Aeromonas*, *Azoarcus*, *Azospira*, *Azospirillum*, *Bradyrhizobium*, *Burkholderia*, *Citrobacter*, *Comamonas*, *Dechloromonas*, *Leptothrix*_Burkholderiales, *Magnetospirillum*, *Pseudomonas*, *Rhodobacter*, *Rhodopseudomonas*, *Shinella*, *Sulfuritalea*, *Thauera*, unclassified_Burkholderiales, *Vibrio*, and unclassified_bacteria (Table S4). Especially, *Azoarcus*, unclassified_bacteria, *Pseudomonas*, *Leptothrix*_Burkholderiales, and *Thauera* accounted for 10.84%, 18.07%, 7.23%, 6.02%, and 6.02% of the keystone aerobic denitrification microbial community.

The correlations between the modules and environmental factors were investigated in Baiyangdian Lake (Figure 6c). The environmental factors T, STP, WAEF-NH_4_^+^-N, WAEF-NO_3_^−^-N, WAEF-N, Res-N, and TN were significantly correlated with the modules and keystone aerobic denitrifying microbial community in Baiyangdian Lake. T exhibited a significantly positive correlation with module 3 (R = 0.68, *p* < 0.01), module 4 (R = 0.68, *p* < 0.01), module 6 (R = 0.68, *p * < 0.01), module 7 (R = 0.68, *p* < 0.01), and module 8 (R = 0.68, *p* < 0.01); STP and WAEF-NO_3_^−^-N exhibited a significantly negative correlation with modules 1–8, and the correlation reached −0.56 to −0.58 (*p* < 0.05) and −0.64 (*p* < 0.05); WAEF-NH_4_^+^-N, Res-N, and TN were positively correlated with modules 1–8, and the correlation reached 0.54–0.64 (*p* < 0.05), 0.65–0.70 (*p* < 0.05), and 0.67–0.71 (*p* < 0.01). WAEF-N was positively correlated with module 1 (R = 0.55, *p* < 0.05), module 2 (R = 0.55, *p* < 0.05), module 5 (R = 0.55, *p* < 0.05), and other modules (R = 0.71, *p* < 0.01). Especially, the keystone aerobic denitrifying microbial community exhibited a significantly positive correlation with T (R = 0.70, *p* < 0.01), WAEF-NH_4_^+^-N (R = 0.58, *p* < 0.05), Res-N (R = 0.64, *p* < 0.05), and TN (R = 0.63, *p* < 0.05) and a significantly negative correlation with STP (R = −0.58, *p* < 0.05). On the basis of all the results, T, STP, WAEF-NH_4_^+^-N, WAEF-NO_3_^−^-N, and TN were important environment factors, which is consistent with the RDA and HP analysis results.

## 4. Conclusions

This is the first report of the characteristics and driving factors of the aerobic denitrifying microbial community, especially, the “keystone species” and the dominant environment factor in natural water ecosystem through the aerobic denitrification functional gene (*nap*A). In this study, the environmental parameters (IEF-N, WAEF-N, SAEF-N, SOEF-N, Res-N, and TN) exhibited significantly spatial differences among the different functional areas, and most of the sediment nitrogen fractions exhibited positive correlations in Baiyangdian Lake. MiSeq revealed a total of 1886 OTUs identified as proteobacteria (42.04–99.08%), unclassified_bacteria (0.92–57.92%), and *Deinococcus-Thermus* (0.00–0.25%). The unclassified genus accounted for an important part in Baiyangdian Lake. The RDA and VIF results showed that T, MC, STP, IEF-NH_4_^+^-N, and WAEF- NH_4_^+^- N were the dominant environment parameters and explained 11%, 13.6%, 27.6%, 16.3%, and 2.8% variation in *α*-diversity. Moreover, the microbial community exhibited significantly spatial differences (R^2^ = 0.4422, *p* < 0.05). T, MC, STP, IEF-NO_3_^−^-N, WAEF-NH_4_^+^-N, WAEF-NO_3_^−^-N, and SAEF-NH_4_^+^-N were the dominant environmental factors and explained the 11.1%, 8.2%, 10.7%, 9.3%, 8.1%, 10.5%, and 7.5% variation of the total variation in the whole aerobic denitrifying microbial community, respectively. The network analysis showed that symbiotic relationships accounted for a major percentage of the microbial networks, and 40.18% nodes belonged to betaproteobacteria. The keystone OTUs belonged to *Comamonas*, *Rhodobacter*, *Achromobacter*, *Aeromonas*, *Azoarcus*, *Leptothrix*_Burkholderiales, *Magnetospirillum*, *Pseudomonas*, *Sulfuritalea*, and *Thauera*. Furthermore, the composition of the keystone aerobic denitrifying microbial community also exhibited significantly spatial differences (R^2^ = 0.4534, *p* < 0.05), and T, STP, IEF-NH_4_^+^-N, IEF-NO_3_^−^-N, WAEF-NO_3_^−^-N, SAEF-NH_4_^+^-N, and TN were the dominant environmental factors. From all the results, this study supplied a new view on investigating the distribution characteristics and driving factors of the aerobic denitrifying microbial community in Baiyangdian Lake.

## Supplementary Materials

Supplementary materials can be found at https://www.mdpi.com/2076-2607/8/5/714/s1.

## Abbreviations

IEF-N	ion-exchangeable form of nitrogen
IEF-NH_4_^+^-N	ion-exchangeable form of ammonia
WAEF-N	weak-acid extractable form of nitrogen
WAEF-NH_4_^+^-N	weak-acid extractable form of ammonia
SAEF-N	strong-alkali extractable form of nitrogen
SAEF-NH_4_^+^-N	strong-alkali extractable form of ammonia
SOEF-N	strong-oxidant extractable form of nitrogen
SOEF-NH_4_^+^-N	strong-oxidant extractable form of ammonia
IEF-TN	ion-exchangeable form of total nitrogen
IEF-NO_3_^−^-N	ion-exchangeable form of nitrate
WAEF-TN	weak-acid extractable form of total nitrogen
WAEF-NO_3_^−^-N	weak-acid extractable form of nitrate
SAEF-TN	strong-alkali extractable form of total nitrogen
SAEF- NO_3_^−^-N	strong-alkali extractable form of nitrate
SOEF-TN	strong-oxidant extractable form of total nitrogen
SOEF-NO_3_^−^-N	strong-oxidant extractable form of nitrate
Res-N	residue nitrogen
TAN	total available nitrogen
TN	total nitrogen
NO_3_^−^-N	nitrate
T	temperature
NO_2_^−^-N	nitrite
MC	Moisture content
NH_4_^+^-N	ammonia
STP	sediment total phosphorus
DO	dissolved oxygen
ORP	oxidation-reduction potential
EC	electrical conductivity
PCoA	principal co-ordinate analysis
adonis	permutational MANOVA
RDA	redundancy analysis
VIF	variance inflation factor
HP	hierarchical partitioning
