# Supplementary material for: Characteristics and Driving Factors of the Aerobic Denitrifying Microbial Community in Baiyangdian Lake, Xiong’an New Area
Source: Microorganisms. 2020 May 11;8(5):714. doi: 10.3390/microorganisms8050714 (PMC7284800; doi:10.3390/microorganisms8050714)
Supplement: Supplementary file 1 [file microorganisms-08-00714-s001.docx]

N fraction introduction:

More information about how the extraction process of the nitrogen forms was performed in the following. As shown in Fig. 1-b, N fractions include: 1) transferable nitrogen forms (i.e., ion exchangeable forms (IEF-N)), 2) weak acid extractable form (WAEF-N), 3) strong alkaline extractable form (SAEF-N), 4) strong oxidant extractable form (SOEF-N), and 5) non-available or residue nitrogen (Res-N). The sum of IEF-N, WAEF-N, SAEF-N, and SOEF-N was defined as total available nitrogen (TAN). The TN of every extract solution was measured, and acted as IEF-TN, WAEF-TN, SAEF-TN, and SOEF-TN. The sum of IEF-TN, WAEF-TN, SAEF-TN, and SOEF-TN was defined as TN of sediment.

Analysis of MiSeq data:

The sequences shorter than 200 base pairs and low-quality sequences (quality score < 25) were removed. To ensure a fair comparison, the size of each sample was normalized to the same sequencing depth by randomly removing the redundant reads. All PCR products were sequenced on an Illumina Miseq Sequencing platform according to standard protocols at Shanghai Majorbio Bio-pharm Technology Co., Ltd. (Shanghai, China).

Network analysis:

Network analysis was performed to investigate the biotic interactions between microbial populations, which indicated how OTUs in the water microbial community interact based on the positive or negative spearman correlations. In this study, a positive correlation implied a mutualistic interaction, whereas a negative correlation indicated competition. The size of each node is proportional to the degree. The thickness of edges is proportional to the spearman correlation (|r|).

Samples site:

In this study, 14 water samples were collected from 5 typical zones in Baiyangdian Lake (Fig. 1-a). In detail, the natural area contained 1 sample sites, including ZZD; breeding area contained 3 sample sites, including FYD, QT, and SHD, respectively; tourist area included 2 sample sites (YYD and SCD); living area existed 4 sample sites, including ZLZ, PYD, FANYD, and BTZXD, respectively; the estuary area included 4 major inflow rivers (PH, BH, FH, and BGYH) with industrial, domestic and agricultural pollution. The TH and ZLH also belonged to the estuary area, however, the analysis of MiSeq data of these samples were not performed successfully, therefore, these samples (TH and ZLH) were not analysed in this study.


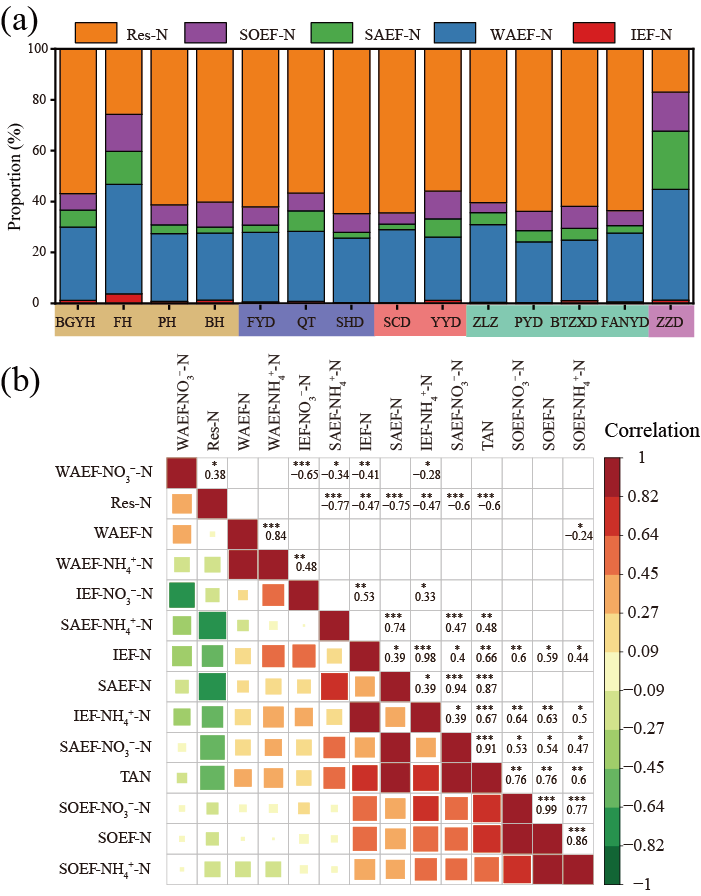


**Figure S1.** Proportion and correlation of different sediment N fractions, and correlation between water water N fractions and sediment N fractions in Baiyangdian Lake. (a), Proportion different sediment N fractions; (b), Correlation of different sediment N fractions.


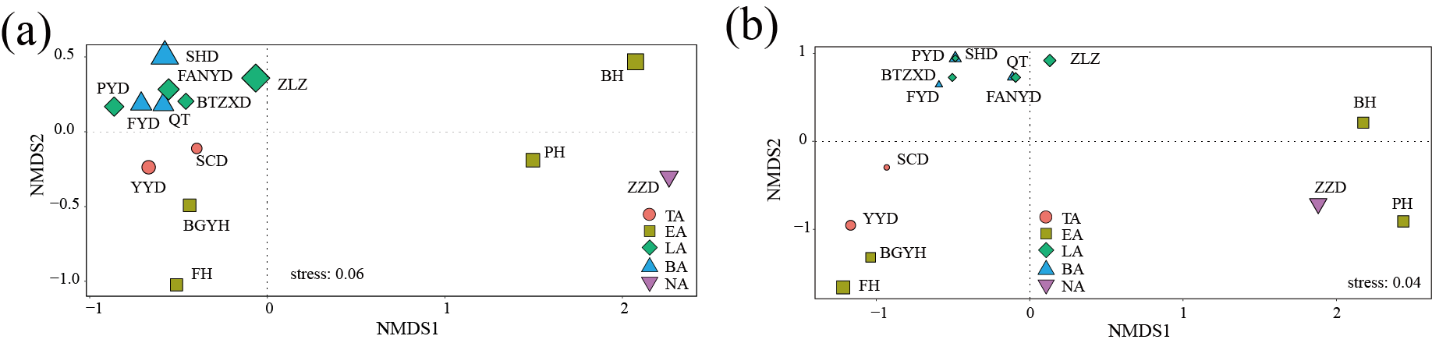


**Figure S2**. NMDS of whole and keystone aerobic denitrifying microbial community in Baiyangdian Lake.

**Table S1.** The information of microbial community diversity and richness estimators in Baiyangdian Lake.

|  | Seq_num | Mean_length | Shannon | Simpson | Pielou | Richness | Chao1 | ACE | Coverage |
| --- | --- | --- | --- | --- | --- | --- | --- | --- | --- |
| BGYH | 14967 | 371.76 | 6.47 | 0.97 | 0.71 | 555 | 663.90 | 665.12 | 0.9913 |
| FH | 14093 | 384.06 | 5.62 | 0.95 | 0.65 | 386 | 485.92 | 492.84 | 0.9922 |
| PH | 17390 | 364.78 | 6.45 | 0.94 | 0.69 | 634 | 677.08 | 677.95 | 0.9952 |
| BH | 18247 | 363.98 | 2.71 | 0.58 | 0.34 | 259 | 292.55 | 304.66 | 0.9968 |
| FYD | 15522 | 372.52 | 5.86 | 0.97 | 0.68 | 383 | 514.10 | 497.37 | 0.9926 |
| QT | 15298 | 373.34 | 6.12 | 0.97 | 0.70 | 419 | 502.06 | 512.80 | 0.9931 |
| SHD | 20379 | 376.52 | 5.67 | 0.95 | 0.67 | 365 | 435.43 | 434.17 | 0.9960 |
| SCD | 11127 | 373.62 | 6.76 | 0.97 | 0.73 | 599 | 762.83 | 766.83 | 0.9842 |
| YYD | 13452 | 376.92 | 6.16 | 0.97 | 0.71 | 391 | 513.35 | 510.74 | 0.9918 |
| ZLZ | 23918 | 366.82 | 7.05 | 0.98 | 0.75 | 708 | 797.65 | 778.93 | 0.9951 |
| PYD | 15207 | 376.24 | 5.34 | 0.95 | 0.65 | 302 | 389.00 | 400.06 | 0.9942 |
| BTZXD | 13214 | 377.62 | 5.90 | 0.95 | 0.68 | 419 | 503.88 | 494.73 | 0.9926 |
| FANYD | 17014 | 373.04 | 5.82 | 0.96 | 0.67 | 415 | 487.79 | 504.66 | 0.9941 |
| ZZD | 13926 | 364.12 | 3.80 | 0.79 | 0.48 | 254 | 290.26 | 292.99 | 0.9962 |

**Table S2.** Community variances explained by environmental factors of microbial community *α*-diversity in Baiyangdian Lake.

|  | VIF | RDA1 | RDA2 | R^2^ |
| --- | --- | --- | --- | --- |
| T | 1.84 | 0.66 | 0.75 | 0.12 |
| MC | 3.27 | 0.70 | 0.72 | 0.37 |
| STP | 6.52 | 1.00 | -0.09 | 0.16 |
| IEF-NH_4_^+^-N | 6.07 | 0.97 | 0.23 | 0.02 |
| IEF-NO_3_^−^-N | 3.88 | -0.19 | 0.98 | 0.06 |
| WAEF-NH_4_^+^-N | 2.23 | 0.80 | 0.60 | 0.10 |
| WAEF-NO_3_^−^-N | 3.18 | -0.11 | 0.99 | 0.10 |
| SAEF-NH_4_^+^-N | 1.82 | 0.22 | -0.98 | 0.37 |
| SAEF-NO_3_^−^-N | 2.19 | 0.20 | 0.98 | 0.32 |

**Table S3.** Community variances explained by environmental factors of whole and keystone aerobic denitrifying microbial community in Baiyangdian Lake.

|  |  | VIF | RDA1 | RDA2 | R^2^ |
| --- | --- | --- | --- | --- | --- |
| Whole aerobic denitrifying microbial community  (*F*=1.70, *P*<0.05) | T | 1.84 | -0.61 | -0.79 | 0.7371 |
|  | MC | 3.27 | -0.76 | 0.65 | 0.2096 |
|  | STP | 6.52 | 0.72 | 0.70 | 0.4596 |
|  | IEF-NH_4_^+^-N | 6.07 | 0.53 | 0.85 | 0.3906 |
|  | IEF-NO_3_^−^-N | 3.88 | 0.09 | 1.00 | 0.3964 |
|  | WAEF-NH_4_^+^-N | 2.23 | -0.69 | 0.73 | 0.2266 |
|  | WAEF-NO_3_^−^-N | 3.18 | -0.95 | -0.33 | 0.3448 |
|  | SAEF-NH_4_^+^-N | 1.82 | 1.00 | 0.04 | 0.2845 |
|  | SAEF-NO_3_^−^-N | 2.19 | -0.48 | 0.87 | 0.1775 |
| keystone aerobic denitrifying microbial community  (*F*=2.76, *P*<0.05) | T | 2.86 | 0.96 | -0.29 | 0.5776 |
|  | MC | 9.85 | 0.49 | 0.87 | 0.1243 |
|  | STP | 10.25 | -0.99 | -0.10 | 0.3414 |
|  | IEF-NH_4_^+^-N | 6.88 | -0.98 | 0.22 | 0.3046 |
|  | IEF-NO_3_^−^-N | 5.70 | -0.69 | 0.72 | 0.3242 |
|  | WAEF-NH_4_^+^-N | 2.83 | 0.35 | 0.94 | 0.1647 |
|  | WAEF-NO_3_^−^-N | 4.95 | 0.93 | 0.37 | 0.3617 |
|  | SAEF-NH_4_^+^-N | 1.90 | -0.68 | -0.74 | 0.2470 |
|  | SAEF-NO_3_^−^-N | 7.67 | -0.17 | 0.99 | 0.1786 |
|  | TN | 8.83 | 0.96 | 0.29 | 0.4168 |

**Table S4.** The information of keystone OTUs in Baiyangdian Lake.

| ID | Abundance | Phylum | Class | Genus | Module | Betweeness centrality | degree |
| --- | --- | --- | --- | --- | --- | --- | --- |
| OTU1892 | 0~1.81% | Proteobacteria | Betaproteobacteria | *Sulfuritalea* | 1 | 6.11 | 14 |
| OTU1883 | 0~0.54% | unclassified | unclassified_d__Bacteria | *unclassified_d__Bacteria* | 1 | 22.86 | 16 |
| OTU1877 | 0~0.14% | Proteobacteria | Alphaproteobacteria | *Rhodopseudomonas* | 1 | 71.17 | 14 |
| OTU1872 | 0~0.54% | unclassified | unclassified_d__Bacteria | *unclassified_d__Bacteria* | 1 | 28 | 9 |
| OTU1855 | 0~4.24% | Proteobacteria | Betaproteobacteria | *Achromobacter* | 1 | 28 | 9 |
| OTU1842 | 0~0.18% | unclassified | unclassified_d__Bacteria | *unclassified_d__Bacteria* | 1 | 4.90 | 16 |
| OTU1840 | 0~0.81% | Proteobacteria | Betaproteobacteria | *unclassified_o__Burkholderiales* | 1 | 0 | 12 |
| OTU716 | 0~0.34% | Proteobacteria | Betaproteobacteria | *Sulfuritalea* | 1 | 100.5 | 10 |
| OTU710 | 0~0.84% | unclassified | unclassified_d__Bacteria | *unclassified_d__Bacteria* | 1 | 0.5 | 9 |
| OTU708 | 0~0.79% | unclassified | unclassified_d__Bacteria | *unclassified_d__Bacteria* | 1 | 4.90 | 16 |
| OTU700 | 0~0.23% | Proteobacteria | Alphaproteobacteria | *Rhodopseudomonas* | 1 | 4.90 | 16 |
| OTU681 | 0~3.25% | Proteobacteria | Gammaproteobacteria | *Pseudomonas* | 1 | 22.86 | 16 |
| OTU666 | 0~16.19% | Proteobacteria | Betaproteobacteria | *Thauera* | 1 | 22.86 | 16 |
| OTU657 | 0~0.49% | Proteobacteria | Betaproteobacteria | *Sulfuritalea* | 1 | 4.90 | 16 |
| OTU656 | 0~2.12% | unclassified | unclassified_d__Bacteria | *unclassified_d__Bacteria* | 1 | 6.11 | 14 |
| OTU647 | 0~7.97% | Proteobacteria | Betaproteobacteria | *unclassified_c_Betaproteobacteria* | 1 | 22.86 | 16 |
| OTU635 | 0~0.96% | unclassified | unclassified_d__Bacteria | *unclassified_d__Bacteria* | 1 | 4.90 | 16 |
| OTU630 | 0~0.35% | Proteobacteria | Betaproteobacteria | *Sulfuritalea* | 1 | 6.11 | 14 |
| OTU165 | 0~0.24% | Proteobacteria | Betaproteobacteria | *Thauera* | 1 | 4.90 | 16 |
| OTU128 | 0~1.11% | Proteobacteria | Betaproteobacteria | *Dechloromonas* | 1 | 4.90 | 16 |
| OTU119 | 0~0.14% | Proteobacteria | Betaproteobacteria | *Ralstonia* | 1 | 6.11 | 14 |
| OTU76 | 0~4.3% | unclassified | unclassified_d__Bacteria | *unclassified_d__Bacteria* | 1 | 6.11 | 14 |
| OTU36 | 0~3.5% | Proteobacteria | Betaproteobacteria | *Azospira* | 1 | 0.5 | 9 |
| OTU570 | 0~3.74% | unclassified | unclassified_d__Bacteria | *unclassified_d__Bacteria* | 2 | 106.17 | 6 |
| OTU1480 | 0~0.82% | Proteobacteria | Betaproteobacteria | *Azoarcus* | 3 | 0 | 7 |
| OTU1452 | 0~0.25% | Proteobacteria | Betaproteobacteria | *Azoarcus* | 3 | 3.57 | 20 |
| OTU1449 | 0~0.41% | Proteobacteria | Gammaproteobacteria | *Pseudomonas* | 3 | 3.57 | 20 |
| OTU1445 | 0~0.19% | Proteobacteria | Alphaproteobacteria | *Rhodopseudomonas* | 3 | 0 | 19 |
| OTU1444 | 0~0.42% | Proteobacteria | Alphaproteobacteria | *Shinella* | 3 | 68.61 | 6 |
| OTU1422 | 0~0.16% | Proteobacteria | Alphaproteobacteria | *Shinella* | 3 | 23.28 | 20 |
| OTU1416 | 0~0.8% | Proteobacteria | Alphaproteobacteria | *Sinorhizobium* | 3 | 3.571 | 20 |
| OTU1409 | 0~0.41% | Proteobacteria | Betaproteobacteria | *Leptothrix_o__Burkholderiales* | 3 | 7.44 | 20 |
| OTU1399 | 0~0.49% | Proteobacteria | Betaproteobacteria | *unclassified_c_Betaproteobacteria* | 3 | 3.57 | 20 |
| OTU1382 | 0~0.35% | Proteobacteria | Betaproteobacteria | *Azoarcus* | 3 | 23.28 | 20 |
| OTU1379 | 0~0.4% | Proteobacteria | Betaproteobacteria | *Azoarcus* | 3 | 3.57 | 20 |
| OTU1361 | 0~0.16% | Proteobacteria | Betaproteobacteria | *unclassified_o__Burkholderiales* | 3 | 7.44 | 20 |
| OTU1353 | 0~12.05% | Proteobacteria | Gammaproteobacteria | *Pseudomonas* | 3 | 32.28 | 22 |
| OTU1343 | 0~0.3% | Proteobacteria | Betaproteobacteria | *Azoarcus* | 3 | 0 | 19 |
| OTU1332 | 0~0.29% | Proteobacteria | Betaproteobacteria | *unclassified_o__Burkholderiales* | 3 | 7.44 | 20 |
| OTU1270 | 0~0.18% | Proteobacteria | Betaproteobacteria | *Thauera* | 3 | 32.28 | 22 |
| OTU1260 | 0~0.24% | Proteobacteria | Betaproteobacteria | *Azoarcus* | 3 | 0 | 19 |
| OTU1250 | 0~0.37% | unclassified | unclassified_d__Bacteria | *unclassified_d__Bacteria* | 3 | 32.28 | 22 |
| OTU1158 | 0~0.28% | Proteobacteria | Gammaproteobacteria | *Pseudomonas* | 3 | 32.28 | 22 |
| OTU1118 | 0~0.22% | Proteobacteria | Betaproteobacteria | *unclassified_o__Burkholderiales* | 3 | 3.57 | 20 |
| OTU1111 | 0~1.75% | Proteobacteria | Alphaproteobacteria | *Azospirillum* | 3 | 23.28 | 20 |
| OTU1054 | 0~0.6% | Proteobacteria | Alphaproteobacteria | *Rhodopseudomonas* | 3 | 3.57 | 20 |
| OTU2230 | 0~4.49% | Proteobacteria | Betaproteobacteria | *Azoarcus* | 4 | 0 | 17 |
| OTU2203 | 0~0.39% | Proteobacteria | Alphaproteobacteria | *Shinella* | 4 | 0 | 17 |
| OTU2191 | 0~0.38% | unclassified | unclassified_d__Bacteria | *unclassified_d__Bacteria* | 4 | 0 | 17 |
| OTU2184 | 0~0.26% | Proteobacteria | Betaproteobacteria | *unclassified_c_Betaproteobacteria* | 4 | 0 | 17 |
| OTU2180 | 0~2.21% | Proteobacteria | Alphaproteobacteria | *Rhodobacter* | 4 | 0 | 17 |
| OTU1303 | 0~0.55% | Proteobacteria | Betaproteobacteria | *Leptothrix_o__Burkholderiales* | 4 | 0 | 17 |
| OTU1271 | 0~0.28% | Proteobacteria | Gammaproteobacteria | *Pseudomonas* | 4 | 0 | 17 |
| OTU1257 | 0~0.66% | Proteobacteria | Alphaproteobacteria | *Bradyrhizobium* | 4 | 0 | 17 |
| OTU1242 | 0~0.66% | Proteobacteria | Betaproteobacteria | *Azoarcus* | 4 | 0 | 17 |
| OTU1225 | 0~0.4% | Proteobacteria | Betaproteobacteria | *Azoarcus* | 4 | 0 | 17 |
| OTU1200 | 0~2.57% | unclassified | unclassified_d__Bacteria | *unclassified_d__Bacteria* | 4 | 0 | 17 |
| OTU1168 | 0~0.78% | Proteobacteria | Alphaproteobacteria | *Rhizobium* | 4 | 0 | 17 |
| OTU1162 | 0~0.61% | Proteobacteria | Betaproteobacteria | *Burkholderia* | 4 | 0 | 17 |
| OTU1145 | 0~0.7% | unclassified | unclassified_d__Bacteria | *unclassified_d__Bacteria* | 4 | 0 | 17 |
| OTU1136 | 0~0.74% | Proteobacteria | Alphaproteobacteria | *Magnetospirillum* | 4 | 0 | 17 |
| OTU1131 | 0~1% | Proteobacteria | Alphaproteobacteria | *Azospirillum* | 4 | 0 | 17 |
| OTU1081 | 0~0.44% | Proteobacteria | Betaproteobacteria | *Leptothrix_o__Burkholderiales* | 4 | 0 | 17 |
| OTU1033 | 0~0.22% | Proteobacteria | Betaproteobacteria | *Thauera* | 4 | 0 | 17 |
| OTU775 | 0~8.72% | Proteobacteria | Gammaproteobacteria | *Aeromonas* | 5 | 43.83 | 7 |
| OTU1435 | 0~1.8% | Proteobacteria | Betaproteobacteria | *Comamonas* | 7 | 0 | 8 |
| OTU1432 | 0~7.26% | Proteobacteria | Gammaproteobacteria | *Citrobacter* | 7 | 0 | 8 |
| OTU1403 | 0~0.43% | Proteobacteria | Alphaproteobacteria | *Ensifer* | 7 | 0 | 8 |
| OTU1390 | 0~0.36% | Proteobacteria | Betaproteobacteria | *Achromobacter* | 7 | 0 | 8 |
| OTU1380 | 0~0.32% | Proteobacteria | Betaproteobacteria | *Achromobacter* | 7 | 0 | 8 |
| OTU1358 | 0~7.38% | Proteobacteria | Betaproteobacteria | *Comamonas* | 7 | 0 | 8 |
| OTU1356 | 0~1.23% | Proteobacteria | Betaproteobacteria | *Comamonas* | 7 | 0 | 8 |
| OTU1347 | 0~1.12% | Proteobacteria | Gammaproteobacteria | *Vibrio* | 7 | 0 | 8 |
| OTU1338 | 0~0.99% | Proteobacteria | Gammaproteobacteria | *Pseudomonas* | 7 | 0 | 8 |
| OTU2185 | 0~1% | Proteobacteria | Alphaproteobacteria | *Magnetospirillum* | 8 | 36.22 | 8 |
| OTU1312 | 0~0.43% | unclassified | unclassified_d__Bacteria | *unclassified_d__Bacteria* | others | 0 | 7 |
| OTU1268 | 0~1.05% | Proteobacteria | Alphaproteobacteria | *Rhodobacter* | others | 0 | 7 |
| OTU1143 | 0~0.41% | Proteobacteria | Betaproteobacteria | *Thauera* | others | 0 | 7 |
| OTU1137 | 0~0.37% | Proteobacteria | Betaproteobacteria | *Leptothrix_o__Burkholderiales* | others | 0 | 7 |
| OTU1130 | 0~0.3% | Proteobacteria | Alphaproteobacteria | *Rhodobacter* | others | 0 | 7 |
| OTU1066 | 0~1.05% | Proteobacteria | Betaproteobacteria | *Ramlibacter* | others | 0 | 7 |
| OTU1042 | 0~0.41% | Proteobacteria | Betaproteobacteria | *Leptothrix_o__Burkholderiales* | others | 0 | 7 |
| OTU1038 | 0~0.67% | unclassified | unclassified | *unclassified* | others | 0 | 7 |
